# Evaluation of quantitative accuracy among different scatter corrections for quantitative bone SPECT/CT imaging

**DOI:** 10.1371/journal.pone.0269542

**Published:** 2022-06-06

**Authors:** Kenta Miwa, Reo Nemoto, Hirotsugu Masuko, Tensho Yamao, Rinya Kobayashi, Noriaki Miyaji, Kosuke Inoue, Hiroya Onodera

**Affiliations:** 1 Department of Radiological Sciences, School of Health Sciences, Fukushima Medical University, Fukushima, Japan; 2 Department of Radiological Sciences, School of Health Sciences, International University of Health and Welfare, Tochigi, Japan; 3 Department of Nuclear Medicine, Cancer Institute Hospital of Japanese Foundation for Cancer Research, Tokyo, Japan; Harvard School of Public Health, UNITED STATES

## Abstract

Although scatter correction improves SPECT image contrast and thus image quality, the effects of quantitation accuracy under various conditions remain unclear. The present study aimed to empirically define the conditions for the optimal scatter correction of quantitative bone SPECT/CT images. Scatter correction was performed by applying dual and triple energy windows (DEW and TEW) with different sub-energy window widths, and effective scatter source estimation (ESSE) to CT-based scatter correction. Scattered radiation was corrected on images acquired using a triple line source (TLSP) phantom and an uniform cylinder phantom. The TLSP consisted of a line source containing 74.0 MBq of ^99m^Tc in the middle, and a background component containing air, water or a K_2_HPO_4_ solution with a density equivalent to that of bone. The sum of all pixels in air, water and the K_2_HPO_4_ solution was measured on SPECT images. Scatter fraction (SF) and normalized mean square error (NMSE) based on counts from the air background as a reference were then calculated to assess quantitative errors due to scatter correction. The uniform cylinder phantom contained the same K_2_HPO_4_ solution and 222.0 MBq of ^99m^Tc. The coefficient of variation (CV) was calculated from the count profile of this phantom to assess the uniformity of SPECT images across scatter correction under various conditions. Both SF and NMSE in SPECT images of phantoms containing water in the background were lower at a TEW sub-window of 3% (TEW3%), than in other scatter corrections, whereas those in K_2_HPO_4_ were lower at a DEW sub-window of 20% (DEW20%). Larger DEW and smaller TEW sub-energy windows allowed more effective correction. The CV of the uniform cylinder phantom, DEW20%, was inferior to all other tested scatter corrections. The quantitative accuracy of bone SPECT images substantially differed according to the method of scatter correction. The optimal scatter correction for quantitative bone SPECT was DEW20% (k = 1), but at the cost of slightly decreased image uniformity.

## Introduction

Bone scintigraphy using ^99m^Tc-labeled phosphate compounds is the most prevalent means of detecting bone metastases of prostate and breast cancer [1]. Planar whole-body bone scintigraphy has high sensitivity, although the specificity is limited to characterizing bone metastases. Adding single photon emission computed tomography (SPECT) to planar acquisition has improved diagnostic confidence [2], and when combined with computed tomography (CT), bone SPECT/CT provides better specificity with more precise localization, and better contrast between hot and cold lesions [3].

Skeletal ^99m^Tc uptake can now be quantified in absolute units (amount of radioactivity per unit volume; kBq/mL), thanks to recent advances in SPECT/CT technology [4]. The use of 3D iterative reconstruction has increased accuracy to within ± 5% of the true radioactivity concentration [5–7]. However, corrections for photon attenuation and scattering, resolution recovery, instrumental dead time, radioactive decay and cross-calibration are usually required to generate precise quantitative SPECT images [8]. Quantitative bone SPECT data provide more objective information that facilitates the discrimination of bone metastases [9], degenerative joint disease [10], medication-related osteonecrosis of the jaw [11] and rheumatic disease [12].

Correction for attenuation and scattered photons has the greatest impact on SPECT image quantitation [7, 13, 14]. Integrated SPECT/CT scanner can correct non-uniform attenuation using CT images. CT acquisition parameters have negligible effect in the accurate attenuation correction of the SPECT images [15]. The CT-based attenuation correction improves the quantitative accuracy of SPECT images [16]. In contrast, scatter correction for quantitative SPECT/CT has not been established. Identifying and subtracting the true number of photons that undergo scatter and lose energy before entering the detector remains a major obstacle to achieving accurate SPECT image quantitation [7, 17].

Scattered counts acquired within photopeak window varies between ~ 25% and 40% in ^99m^Tc imaging, which leads to deteriorated image contrast and poor SPECT image quantitation [18]. The most popular methods of correcting scatter in SPECT images are usually based on energy window-based scatter correction (EWSC), the simplest of which use dual (DEW) or triple (TEW) energy windows [19, 20]. Empirical and simulated studies using ^99m^Tc have found that DEW and TEW improve contrast rather than quantitative accuracy in SPECT images [21–23]. Previous studies reported that contrast and image resolution were considerably improved after DEW and TEW so physicians could look at defects better in ^99m^Tc cardiac perfusion SPECT images [24, 25]. However, differences in methods of scatter correction and among conditions such as the width of sub-energy windows can lead to over- or under-correction that increases image noise and results in poor quantitation [26]. The appropriate determination of sub-energy window width on energy spectra should be optimal to improve bone SPECT image quality and quantitative accuracy [27]. Moreover, effective scatter source estimation (ESSE) is combined with measured transmission data for commercial scatter correction [28]. This is because an effective scatter source is estimated for each projection view based on object-independent scatter kernels obtained using Monte Carlo simulations [29].

Although all scatter correction methods should improve contrast, and thus the quality of SPECT images, the effect of quantitation accuracy under various conditions remains unclear. Phantoms are useful and essential to define the characteristics of the quantitative accuracy of scatter correction that cannot be determined in clinical studies because they can be constructed with known materials and desirable activity concentrations. The present study aimed to empirically define the optimal conditions for correcting scatter on quantitative bone SPECT/CT images by measuring the quantitative accuracy of correction methods under various conditions using phantoms.

## Materials and methods

### Data acquisition and image reconstruction

All imaging data were acquired using a Brightview XCT SPECT/CT system (Philips Healthcare, Cleveland, OH, USA) with a high-resolution flat panel x-ray detector (40 × 30 cm^2^) with low-dose cone-beam CT imaging for localization, and attenuation correction of images. The detector is mounted on the same gantry as the SPECT camera, which allows the acquisition of SPECT and cone-beam CT images. The SPECT images were acquired under a ± 10% main energy window at 140 keV with ⅜″ crystal thickness, a low-energy high-resolution collimator (LEHR), 128 × 128 matrix with 4.8-mm pixels, and 60 projections of 20 s/view over 360° in circular orbit continuous acquisition mode. The Brightview XCT SPECT/CT system allows the simultaneous setting of 16 energy windows. With a 360° rotation of the gantry, a 47-cm diameter transverse field of view (FOV) and a 14.4-cm axial length can be visualized along a patient during slow rotation (60 s per rotation) as a cone-beam CT image [30]. We reconstructed the SPECT images using the Philips Astonish (Philips Healthcare) 3D iterative method, with combinations of 15 subsets and 2 iterations, attenuation, scatter correction, and resolution recovery.

### Phantoms

**Fig 1:**
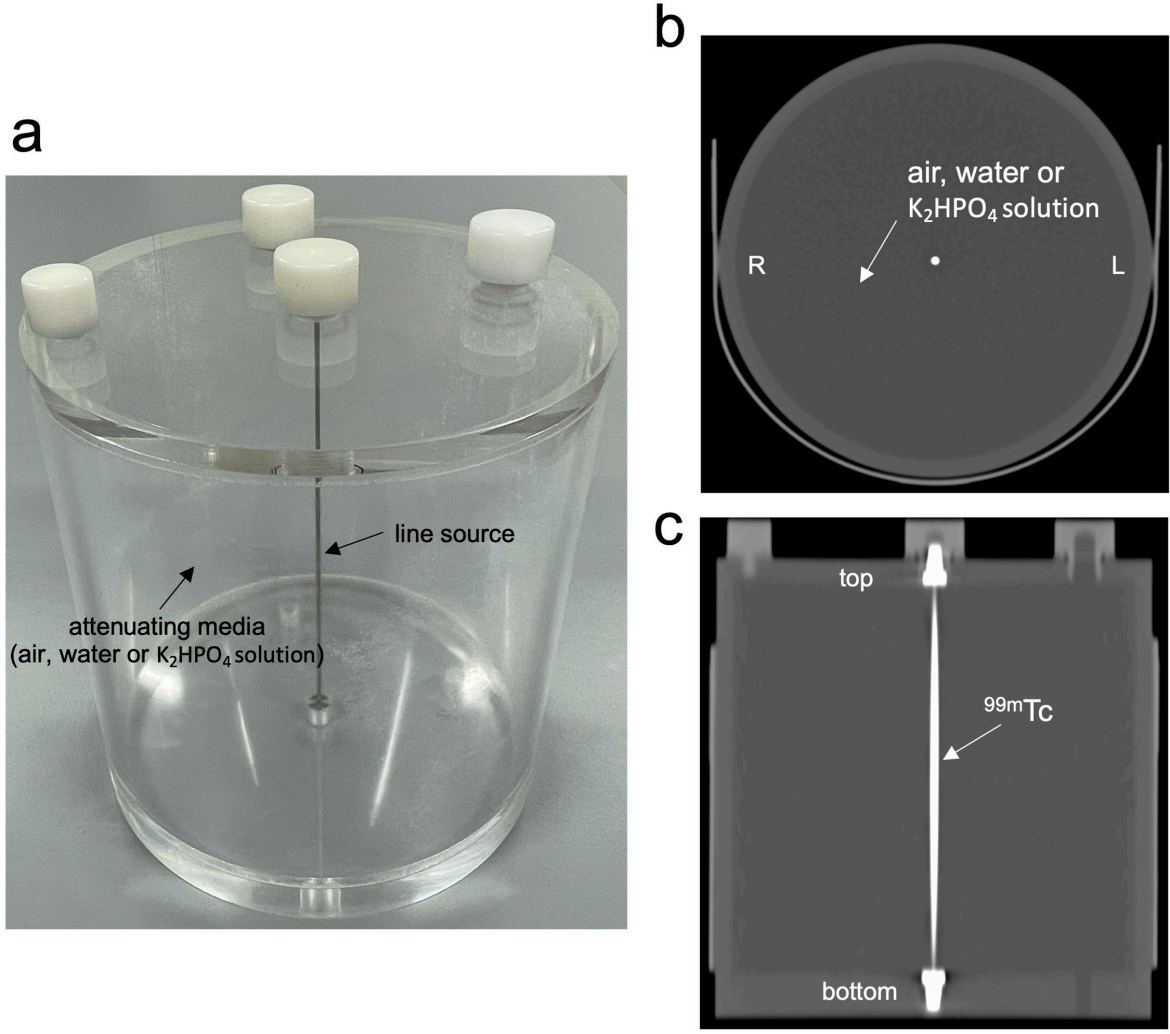
Setup of NEMA SPECT triple line source phantom. Overview (a), and CT axial (b) and coronal (c) views of triple line source phantom.

The inner diameter and inner length of a NEMA SPECT Triple Line Source Phantom (TLSP; Data Spectrum Corp., Durham, NC, USA) were both 20 cm and those of an uniform cylinder phantom were 16 and 15 cm, respectively. The TLSP consisted of triple line sources with a diameter of ~ 1 mm and a height of 18.4 cm. We removed two of the three line sources, leaving the remaining source in the center (Fig 1). Thus, the TLSP consisted of the line source containing 74.0 MBq of ^99m^Tc at the center of the phantom where the attenuating medium was air (reference), water or a K_2_HPO_4_ solution with a density equivalent to that of bone. The uniform cylinder phantom contained the same K_2_HPO_4_ and 222.0 MBq of ^99m^Tc.

### Scatter correction

**Fig 2:**
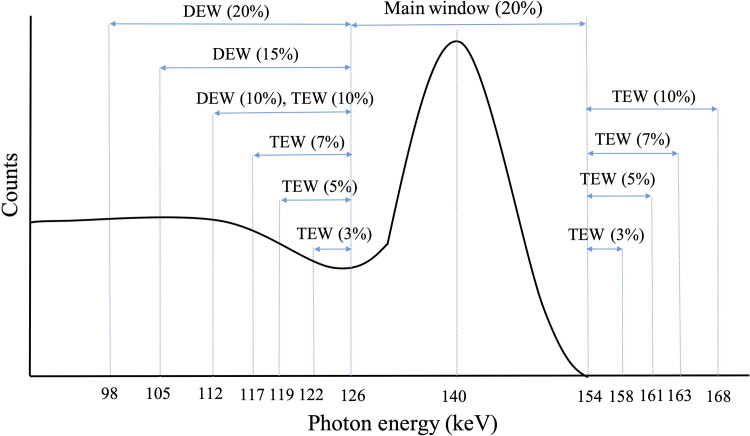
Acquired energy windows.

We applied the following scatter correction methods and the energy window settings for EWSC shown in Table 1 and Fig 2.

**Table 1:** Overview of energy window widths and scatter correction factors for energy window scatter correction.

Scatter correction	Main window (%)	Sub window (%)	*k* value
DEW	±10	5	1.0
		10	
		15	
		20	
TEW	±10	3	3.3
		5	2.0
		7	1.4
		10	1.0

DEW, dual-energy window; ESSE, Effective scatter source estimation; TEW, triple energy window.

#### Dual-Energy Window (DEW)

Data were simultaneously recorded within main energy window and within a secondary lower Compton sub-window in DEW. The spatial distribution of the scattered radiation acquired in the Compton window is considered a good estimate of the distribution of scattered radiation in the main energy window [31]. We applied 5%, 10%, 15%, 20% Compton sub-windows. The correction consisted of subtracting a fraction of the Compton data from the photopeak data as follows [19] (Eq 1):

$${\text{C}}_{prim}={\text{C}}_{total}-k\cdot {\text{C}}_{s}$$ (1)

where *C*_*prim*_ is the estimated unscattered radiation counts in the photopeak window, *C*_*total*_ is the total counts in the photopeak window, and *C*_*s*_ is the total counts in the Compton window. The scatter multiplier (*k*) has been determined heuristically for our SPECT acquisition system and reconstruction algorithm. Koral et al. and Luo et al. reported that the best agreement for experimental, compared with predicted counts in ^99m^Tc images is k = 1.0 rather than 0.5. Therefore, we applied k = 1.0 in the DEW [32–34].

#### Triple Energy Window (TEW)

The TEW includes two small sub-windows around the main energy window that are used to estimate the amount of scatter in the main energy window. Because various widths of energy sub-windows have been applied [20, 35], we investigated the effects of sub-windows with widths of 3%, 5%, 7%, 10%. Scatter correction was calculated as follows (Eqs 2 and 3):

$${\text{C}}_{prim}={\text{C}}_{total}-\frac{1}{2}\left(k_{lower}\times C_{lower}-k_{higher}\times C_{higher}\right)$$ (2)

$$\text{k}=\frac{{\text{W}}_{prim}}{W_{sub}}$$ (3)

where *C*_*prim*_ is the estimated count of unscattered radiation in the photopeak window, *C*_*total*_ is the total count in the photopeak window, *k*_*lower*_ and *k*_*higher*_ are the respective multiplication factors, and *C*_*lower*_ and *C*_*higher*_ are counts in the lower and higher energy sub-windows, respectively, and *W*_*prim*_ and *W*_*sub*_ are widths (in keV) of the photopeak and energy sub-windows, respectively.

#### Effective Scatter Source Estimation (ESSE)

The ESSE is the standard Philips method of correcting scatter [28, 32]. Briefly, this method produces an effective scatter source obtained by convolving the radioactivity distribution with several kernels [36]. The effective scatter source kernel and the relative scatter attenuation coefficient kernel for convolution are derived from Monte Carlo simulations and depend on photon energy [37]. Scatter projection data are then estimated by forward-projecting the effective scatter source.

### Data analysis

A circular region of interest (ROI) was placed at the center of the TLSP. The sum of all pixel counts per slice in air, water and the K_2_HPO_4_ solution were measured on SPECT images generated by the TLSP. The counts per slice were acquired from the average of the counts in 10 slices. The scatter fraction (SF) and normalized mean square error (NMSE) based on counts from the air background as the reference were calculated to assess quantitative errors due to scatter correction as follows (Eqs 4 and 5):

$$SF\;\left(\%\right)=\frac{\left(T_{water\;or\;bone}-T_{air}\right)}{T_{water\;or\;bone}}\times 100\;\left(\%\right)$$ (4)

$${\text{NMSE}}_{ROI}\;\left(\text{\%}\right)=\frac{\sum \sum {\left|T_{air}-T_{water\;or\;bone}\right|}^{2}}{\sum \sum {\left|T_{air}\right|}^{2}}\times 100\;\left(\%\right)$$ (5)

where T_air_ and T_water or bone_ were the average counts of the ROI in the phantoms containing air and in that containing water or bone equivalent solution, respectively.

The coefficient of variation (CV) was evaluated at an 80% circular ROI placed at the center of an uniform cylinder phantom. We calculated the CV from the count profile of this phantom to assess SPECT image uniformity across all scatter corrections as follows (Eq 6):

$$CV\left(\%\right)=SD/mean\times 100$$ (6)

where *SD* and *mean* are the standard deviation and means of counts, respectively, in different scatter corrections. The CV represents the amount of statistical noise in SPECT images, which reflects uniformity after scatter correction.

## Results

**Fig 3:**
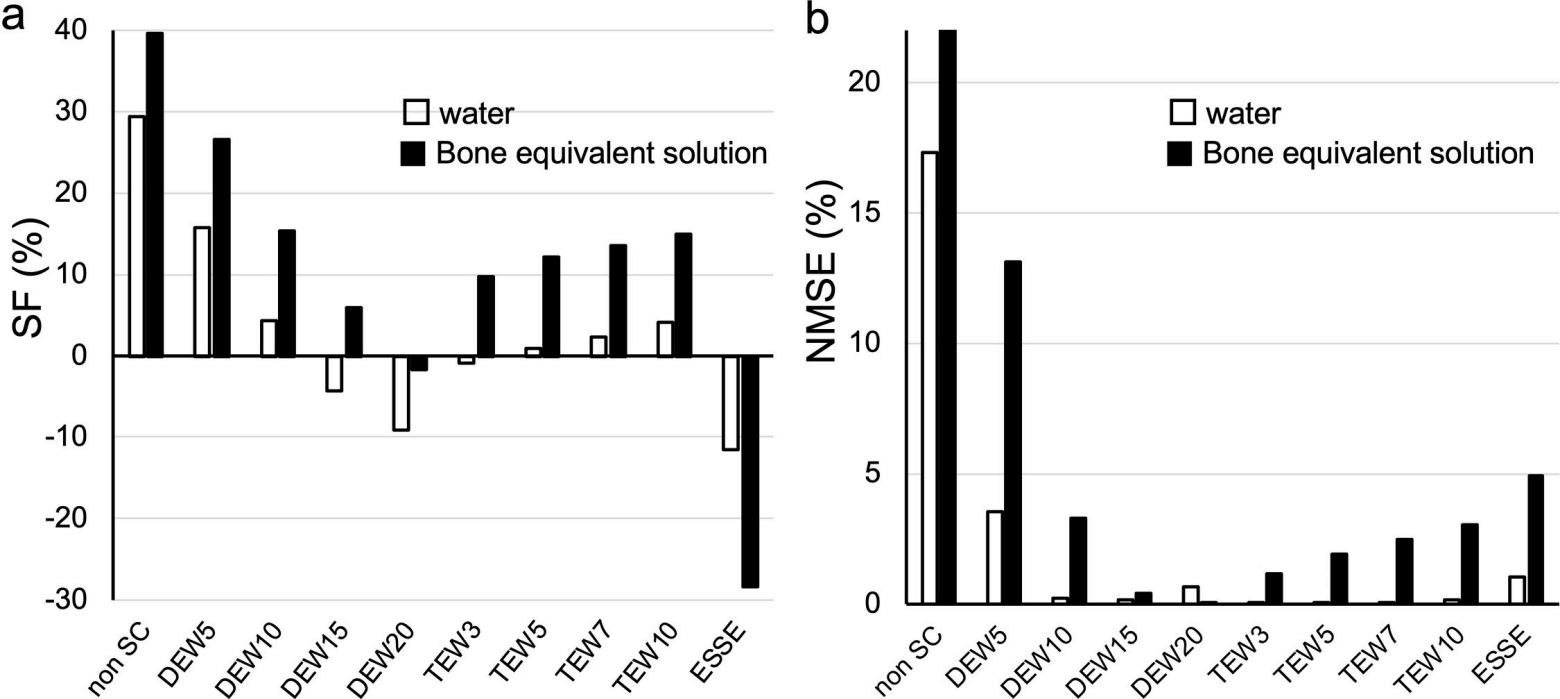
Scatter fraction (a) and normalized mean-square error (b) of SPECT images after scatter correction in phantoms containing water or bone equivalent solution. DEW 5, 10, 15, 20, are dual energy window sub-windows of 5%, 10%, 15%, 20%; ESSE, effective scatter source estimation; non SC, no scatter correction; TEW 3, 5, 7, 10 are triple energy window sub-windows of 3%, 5%, 7%, 10%.

**Fig 4:**
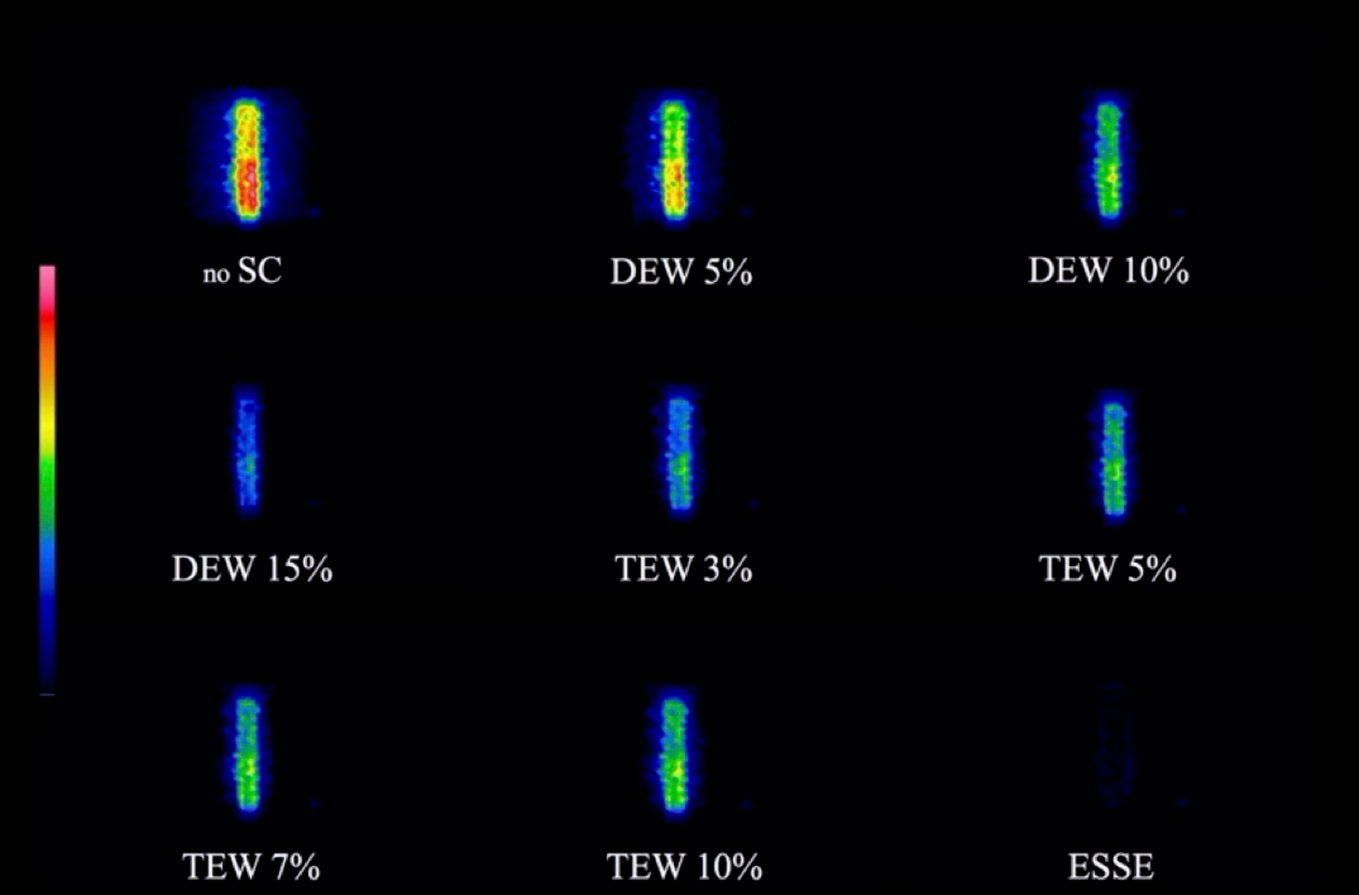
Images of scattered radiation after scatter correction. Increased red intensity indicates more residual scattered radiation. DEW, dual energy window; ESSE, effective scatter source estimation; SC, scatter correction; TEW, triple energy window.

Fig 3A and 3B shows the SF and NMSE, respectively, for each scatter correction. Both SF and NMSE in SPECT images of phantoms containing water in the background were lower at a TEW sub-window of 3% (TEW3%) than any other scatter correction, whereas those in the K_2_HPO_4_ solution were lower at a DEW sub-window of 20% (DEW20%). The SF and NMSE became smaller with larger DEW and smaller TEW sub-energy windows, and ESSE overcorrected the scatter radiation in the K_2_HPO_4_ solution. Table 2 shows the CV of the uniform cylinder phantom. The CV of ESSE was superior, whereas the CV of DEW20% was inferior to that of any other assessed method of scatter correction. We visually confirmed the ability of different scatter correction technologies in scatter radiation images after scatter correction (Fig 4). Scattered radiation images were obtained by subtracting the reference image with DEW20% (considered as the most accurate in bone equivalent solutions) from the SPECT images with scatter corrected under different conditions. The remaining scattered radiation was visually almost identical between DEW20% and ESSE, although the scattered radiation of ESSE might be overcorrected.

**Table 2:** Coefficients of variation at center of uniform cylinder phantom.

Scatter corrections	CV
None	5.27
DEW5%	3.93
DEW10%	3.79
DEW15%	4.71
DEW20%	6.97
TEW3%	4.14
TEW5%	3.95
TEW7%	3.94
TEW10%	3.74
ESSE	3.48

CV, coefficient of variation; DEW, dual energy window; ESSE, effective scatter source estimation; TEW, triple energy window.

## Discussion

Scatter correction for quantitative bone SPECT images has not yet been standardized. We evaluated various scatter correction methods based on EWSC and ESSE using phantoms. Our findings indicated that the accuracy of scatter correction for SPECT image quality and quantitation depends on the method applied, sub-energy window width and the background material that produces scattered radiation. We also found that the scatter correction for quantitative bone SPECT was optimal with a DEW20% sub-window (k = 1).

We applied a K_2_HPO_4_ solution with a density equivalent to that of bone [38, 39]. The trends of SF and NMSE between water and the K_2_HPO_4_ solution in the background component of the phantom considerably differed (Fig 3). The effects of photon scatter by bone and the accuracy of scatter correction for bone SPECT/CT images in clinical practice can be simulated and investigated using a bone equivalent solution. Miyaji et al. [40] validated the accuracy of a new reconstruction method for bone SPECT/CT (xSPECT Bone; Siemens Healthineers), using a K_2_HPO_4_ solution, and Yoshii et al. [41] developed a new phantom containing a K_2_HPO_4_ solution that allows consideration of scatter and photon attenuation due to bone in ^18^F-NaF PET/CT images.

Both SF and NMSE in SPECT images of phantoms containing background K_2_HPO_4_ solution were lower at DEW15% or DEW20% than with TEW scatter correction (Fig 3). An experimental study of the effects of backscattered radiation from various materials and ^99m^Tc radionuclide showed that photons were backscattered in energy regions below ~ 120 keV and did not affect the photopeak [22]. We considered that DEW method, which contained enough multi-forward and backscattered radiation due to bone in the sub-energy window, would be an appropriate choice for scatter correction. Several studies using Monte Carlo simulation [27, 42, 43] and clinical data [7, 44] have found that DEW method with an optimized sub-energy window width and *k* value offered the most appropriate scatter correction of ^99m^Tc SPECT images in terms of improved contrast, higher SNR and good quantitation.

The SF and NMSE decreased with wider DEW sub-energy windows. However, CV was higher in DEW sub-window 20% (Table 2). A wide sub-energy window can measure largely radiations with large deflection angle and higher order scatter. The correction can improve image contrast and quantitation by removing too many photons far from the actual source location, but not enough at the source location. Thus, image uniformity in SPECT images were considered to be slightly degraded [18]. Moreover, DEW and TEW assume that the sub-energy window of scattered radiation are minimally contaminated by unscattered radiations, and the presence of unscattered radiations in the sub-energy window leads to an overestimate of the scatter. Subtraction of this estimate thus reduces scatter radiation in the photopeak, but also reduces the primary signal and thereby increases the noise (CV) of the scatter corrected data [25].

Although the SPECT image corrected using ESSE appeared essentially identical to that of DEW20% sub-window, the SF of ESSE was overestimated in SPECT images acquired from phantoms containing water and background K_2_HPO_4_ solution. This might be due to errors specific to our phantom. The ESSE generates good results in a wide range of application [31, 32, 45, 46] and improves image contrast as well as the quantitative accuracy of radioactivity estimation. However, our findings were inconsistent with these results. The large attenuation between the source and the point of Compton interaction in the large phantom used herein might have resulted in slight errors in the calculation of an effective scatter source for ESSE. Moreover, the CT value of the background might be higher than actual bone due to the high concentration of K_2_HPO_4_ in the solution.

The present study has several limitations. We analyzed data generated by a phantom simulating patient-specific bone. Further studies should investigate bone ^99m^Tc SPECT images acquired from actual patients. We validated only scatter correction in SPECT images for quantitative accuracy. Most corrections such as photon attenuation and scattering, as well as resolution recovery influence quantitative SPECT images [7]. The combination effects of various correction methods in quantitative SPECT images requires further investigation.

## Conclusions

The quantitative accuracy of bone SPECT images considerably differed according to the method of scatter correction. The optimal scatter correction for quantitative bone SPECT was DEW20% (k = 1) according to our phantom study using water and K_2_HPO_4_ solution as scatter radiation components representing soft tissue or bone, respectively, but at the cost of slightly decreased image uniformity. The present findings provide useful information about how to confirm optimal scatter correction for quantitative evaluations of bone SPECT images. Compton scatter is object-dependent and spatially varying. Further studies should validate bone ^99m^Tc SPECT images acquired from more complex geometry and realistic phantom, e.g. NEMA IEC body phantom, and actual patients using optimal scatter correction obtained from our phantom study.
